# Cine MRI-Derived Radiomics for the Detection of Functional Tricuspid Regurgitation in Pulmonary Hypertension: A Proof-of-Concept Study

**DOI:** 10.3390/jcdd12090353

**Published:** 2025-09-13

**Authors:** Kai Lin, Roberto Sarnari, Daniel Z. Gordon, Michael Markl, James C. Carr

**Affiliations:** Department of Radiology, Northwestern University Feinberg School of Medicine, 737 N Michigan Ave, Ste 1600, Chicago, IL 60611, USA

**Keywords:** functional tricuspid regurgitation, Cine MRI, radiomics

## Abstract

(1) Objective: The objective was to test the hypothesis that cine MRI-derived radiomic features can detect functional tricuspid regurgitation (FTR) in the context of pulmonary hypertension (PH). (2) Materials and methods: In total, 53 PH patients were retrospectively enrolled. Thirty-three patients had echocardiography-defined mild-to-severe FTR, while the other twenty patients had no or trivial regurgitation. For all participants, 93 radiomic features were extracted from four-chamber cine MRI using a fixed-size region of interest (ROI) located in the right atrium (RA), 0.5–1 cm above the tricuspid valve. The levels of radiomic features were averaged over the ventricular systole and compared between patients with and without FTR using t tests. In patients with FTR, radiomic features were related to hemodynamic parameters in the right heart using the Pearson correlation coefficient (r). (3) Results: There were no significant differences in demographic information, right heart catheterization (RHC) results, and most cine MRI-derived cardiac function indices between the two subject groups. Eight of ninety-three radiomic features were significantly different between PH patients with and without FTR. Radiomic features can be used to discriminate two subject groups (AUC = 0.77). In patients with FTR, multiple radiomic features are related to the pressure in the RA, right ventricle (RV), and pressure difference between RA and RV (r: 0.4 to 0.55), *p* values < 0.05. (4) Conclusion: Cine MRI-derived radiomic features of the cardiac blood pool differ between PH patients with and without FTR. Cine MRI shows promise as a method for assessing FTR in the context of complex cardiovascular diseases (CVDs).

## 1. Introduction

Functional tricuspid regurgitation (FTR) occurs when the tricuspid valve closes improperly, primarily due to conditions affecting structures outside the leaflets or apparatus itself [1]. FTR is a common comorbidity in pulmonary hypertension (PH), as elevated right-sided pressures lead to right ventricular (RV) dilation and subsequent annular enlargement of the tricuspid valve. This enlargement prevents proper tricuspid leaflet coaptation during systole, resulting in regurgitation. Conversely, FTR can exacerbate PH by creating a vicious cycle of worsening RV dysfunction and systemic venous congestion [2]. Therefore, assessing FTR is critical in PH management, as it provides key insights into disease severity, involvement, and prognosis.

Although echocardiography is a primary tool for assessing FTR in clinical practice, it has limitations. For instance, the width of the vena contracta (VC) is a commonly used, guideline-recommended echocardiographic index for diagnosing and grading FTR [3]. However, the shape of the regurgitation jet can be irregular, and VC measurements vary significantly across different imaging planes. Additionally, VC widths can be affected by technical and physiological factors, such as machine settings and flow rate [4]. Another frequently used echocardiographic index, the proximal isovelocity surface area (PISA), has been found to underestimate the severity of FTR in 20% to 30% of patients [5].

With a large field of view (FOV), good contrast between the myocardium and blood pool, and reduced operator dependence, cardiac magnetic resonance imaging (MRI) has become a promising tool for the description of FTR. On regular cine MRI (with bright blood techniques), semi-quantitative assessment of FTR can be performed by visualizing an area of signal drop above the tricuspid valve due to “transvers spins” brought on by flow turbulences resulting from the regurgitation [6]. However, the detection and grading of subtle changes in “dark” or “bright” on cine images based on the “naked eye” can be challenging. Therefore, a more objective method for detecting signal changes in cine MRI is required for directly detecting and quantifying FTR.

Our previous studies have shown that cine MRI-derived radiomic features obtained from the cardiac blood pool and great vessels can represent cardiac structural, functional, and hemodynamic changes in the context of PH [7,8,9]. However, data are lacking on whether this method can also be used to describe valve regurgitation in PH. To address this critical “piece” of the “puzzle” regarding the application of cine MRI in PH management, we compared radiomic features acquired from the right atrium (RA) in PH patients with and without FTR. The aim of this study was to test the hypothesis that cine MRI-derived radiomic features can detect FTR in the context of PH.

## 2. Materials and Methods

### 2.1. Patient Population

With the approval of the institutional review board (IRB), 53 consecutive PH patients (23 males, 36–89 years old) who underwent MRI, echocardiography, and right heart catheterization (RHC) were retrospectively enrolled. PH was defined as a mean pulmonary artery pressure (mPAP) > 20 mmHg measured with RHC [10]. All participants provided written informed consent before MRI examinations.

The inclusion criteria included the following: (1) age 18–89 years.

The exclusion criteria included the following: (1) presence of a permanent pacemaker or cardioverter-defibrillator; (2) primary valve diseases or congenital heart diseases; (3) prior stroke, neurodegenerative disorders, mental health problems, or cancerous malignances; or (4) other contraindications to cardiac MRI, such as implanted metal devices or claustrophobia.

Demographic data, including age, sex, height, weight, and body mass index (BMI), were collected before MRI scans.

### 2.2. Cine MRI Protocol

Cardiac MRI examinations were performed on a 1.5 T scanner (Magnetom, Aera, SIEMENS, Erlangen, Germany) using a fixed protocol. After a three-plane fast localization pulse sequence was applied to find the anatomic orientation of the entire scan, a segmented balanced steady-state free precession (bSSFP) sequence was employed in the two-chamber, three-chamber, four-chamber, and short-axis orientations to acquire cine images. The imaging parameters were as follows: repetition time (TR)/echo time (TE) = 43.4/1.1 ms; echo spacing = 2.71 ms; segment = 16; flip angle = 65°; field of view (FOV) = 360 × 360 mm^2^; basal resolution = 224; slice thickness = 6 mm; gap = 4 mm; bandwidth = 930 Hz/pixel; generalized autocalibrating partially parallel acquisition (GRAPPA) technique was applied with a reduction factor R = 2. Each myocardial slice was acquired during a breath hold (at end-expiration) using retrospective electrocardiogram (ECG) gating (with 25 retrospectively constructed cardiac phases). Ten to twelve short-axis slices were acquired to cover the entire left/right ventricles (LV/RV) from base to apex.

### 2.3. Cardiac Function and Mass Analysis for LV and RV

Cine images were processed on a dedicated workstation affiliated with the MRI scanner by an experienced operator (reviewer #1, 18 years of experience in cardiovascular imaging) using a standard clinical workflow with Argus software (Siemens, Erlangen, Germany). For each slice and time phase, the epicardial and endocardial borders of the LV and RV were traced manually. LV and RV volumes at each cardiac phase were obtained by summing the LV areas of all slices from the base to the apex of the ventricles. The end-diastolic volume (LVEDV and RVEDV), end-systolic volume (LVESV and RVESV), stroke volume (LVSV and RVSV), cardiac output (LVCO and RVCO), cardiac index (LVCI and RVCI), ejection fraction (LVEF and RVEF), and LV mass (LVM) were then calculated. Myocardial density was assumed to be 1.05 g/mL [11].

### 2.4. Echocardiography as a Part of “Standard of Care” and Definition of the Existence of FTR

Comprehensive 2D and Doppler echocardiography examinations were performed in our institution using an ultrasound machine (Vivid E9, GE Healthcare, General Electric Corp., Waukesha, WI, USA). Two-dimensional images were acquired in the parasternal long-axis and short-axis views, three standard apical views, and RV-focused views during three consecutive cardiac cycles. Images were digitally stored at 50–90 frames/second. The chamber dimensions were measured using the 2D-guided M-mode method. E, A, E/A, mitral deceleration time (DT), and tricuspid regurgitation velocity were obtained. The patients with “no or trivial regurgitation” in tricuspid valve were defined as “without FTR”. Patients with mild (VC width < 3 mm), moderate (VC width between 3 and 7mm), or severe (VC width > 7mm) regurgitation were defined as “with FTR” [4].

### 2.5. Right Heart Catheterization (RHC)

Patients were examined in the fasted state after sedation by interventional cardiologists. In the supine position, standard RHC was performed through a 7–9 F sheath via the internal jugular or femoral vein. A Swan–Ganz catheter was then inserted. Pressures in the RA, RV, systolic, diastolic, and mean pulmonary artery (sPAP, dPAP, mPAP), and pulmonary capillary wedge pressure (PCWP) were measured at end-expiration from continuous recordings of pressure tracings digitized at 240 Hz, and represent the mean of ≥ 3 beats. Pulmonary vascular resistance (PVR) was calculated as (mPAP—PCWP)/LVCO.

### 2.6. Radiomic Feature Extraction at Ventricular Systole

**Figure 1:**
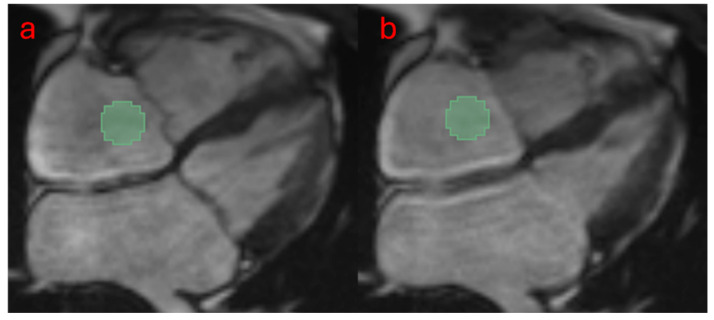
The workflow for extracting radiomic features is as follows: A male PH patient with moderate tricuspid regurgitation was analyzed. A fixed-size ROI (3% of the image, 101 voxels) was placed in the RA (0.5–1 cm above the tricuspid valve) to extract radiomic features. A total of eight images were included, spanning from the start of ventricular systole (panel **a**) to the end of systole (panel **b**). The values of each feature obtained from these images were averaged for quantitative analysis.

Cine MRI images acquired with a four-chamber view during 25 retrospective phases were reviewed. Images during the period of ventricular systole were analyzed with 3D Slicer software (Version 5.03, http://www.slicer.org) [12]. A round region of interest (ROI) was positioned at the RA (0.5–1 cm above the tricuspid valve) in each image (phase) using the segmentation tool embedded in 3D Slicer, accounting for 3% of the entire field of view (FOV) [9]. See Figure 1. To avoid the influence of the size of ROI, this ROI had 101 voxels. In total, 93 radiomic features were extracted from the ROI and entered quantitative analysis, including first-order statistics (18 features), gray level cooccurrence matrix (GLCM, 24 features), gray level dependence matrix (GLDM, 14 features), gray level run length matrix (GLRLM, 16 features), gray level size zone matrix (GLSZM, 16 features), and neighboring gray tone difference matrix (NGTDM, 5 features). These features were extracted using the PyRadiomics plug-in [7]. Shape-related features were excluded because of the same round shape fixed-size ROI in all images.

Reader #2 performed radiomics analysis independently using the same workflow to test the interobserver variabilities of radiomic feature extraction. Reader #1 reanalyzed the cases a week later to test the intraobserver variabilities.

### 2.7. Statistical Analysis

All continuous variables are represented as the mean ± one standard deviation (SD). Since FTR mostly happens during ventricular systole, levels of radiomic features among multiple systolic phases were averaged and compared between PH patients with and without FTR using t tests. Receiver operating characteristic (ROC) curves and the area under the curve (AUC) were used to estimate the capability of individual radiomic features for discriminating PH patients with and without FTR. The Pearson correlation coefficient (r) was used to assess the correlations between radiomic features and hemodynamic parameters. An r > 0.4 was defined as a moderate correlation. A *p* value < 0.05 was defined as “significant”. Good intraobserver and interobserver variances were defined by the intraclass correlation coefficient (ICC) > 0.7 and coefficient of variance (CoV) < 20%.

## 3. Results

Cine images of all 53 cases were eligible for quantitative analysis. Figure 1 shows the workflow of data processing. Radiomic features of the RA were successfully extracted from all cases. There were no significant differences in demographic information, RHC results, and most cine MRI-derived cardiac function indices between the two subject groups. See Table 1.

**Figure 2:**
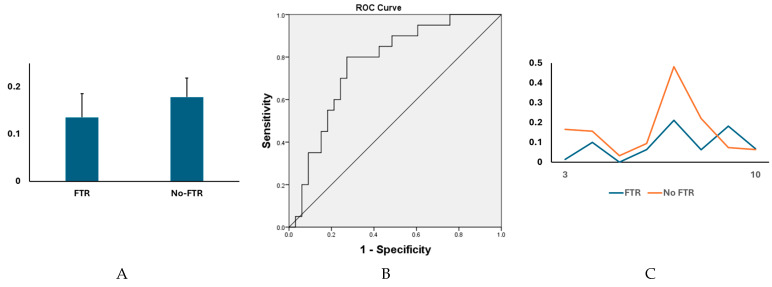
There were significant differences in multiple cine MRI-derived radiomic features between PH patients with and without FTR. (**A**) The levels of “Small Area Low Gray Level Emphasis” (GLSZM) were significantly different between two subject groups (0.14 ± 0.05 vs. 0.18 ± 0.04, *p* = 0.003). (**B**) The levels of “Small Area Low Gray Level Emphasis” (GLSZM) could efficiently discriminate the two subject groups (AUC = 0.77, 95% confident interval [CI] = 0.71–0.83, *p* < 0.05). (**C**) A head-to-head comparison between a postcapillary patient with moderate-to-severe FTR (female, 54 years) and a postcapillary patient with no FTR (female, 64 years). The levels of “Small Area Low Gray Level Emphasis” (GLSZM) acquired through systole (phases 4 to 11 of cine images).

Eight of 93 radiomic features were significantly different between PH patients with (*n* = 33) and without (*n* = 20) FTR, including “Range” (First order) (66.6 ± 22.8 vs. 54.9 ± 14.7, *p* = 0.044), “Contrast” (GLCM) (0.34 ± 0.13 vs. 0.27 ± 0.08, *p* = 0.045), “Difference Average” (GLCM) (0.32 ± 0.1 vs. 0.27 ± 0.08, *p* = 0.047), “Difference Variance” (GLCM) (0.22 ± 0.06 vs. 0.19 ± 0.04, *p* = 0.043), “Run Length NonUniformity” (GLRLM) (12 ± 4.3 vs. 9.7 ± 3.3, *p* = 0.045), “Large Area High Gray Level Emphasis” (GLSZM) (3317 ± 1071 vs. 4078 ± 1328, *p* = 0.026), “Low Gray Level Zone Emphasis” (GLSZM) (0.4 ± 0.09 vs. 0.46 ± 0.1, *p* = 0.03), and “Small Area Low Gray Level Emphasis” (GLSZM) (0.14 ± 0.05 vs. 0.18 ± 0.04, *p* = 0.003). ROC curves showed that “Small Area Low Gray Level Emphasis” can be used to discriminate two subject groups (AUC = 0.77). See Figure 2A,B.

**Figure 3:**
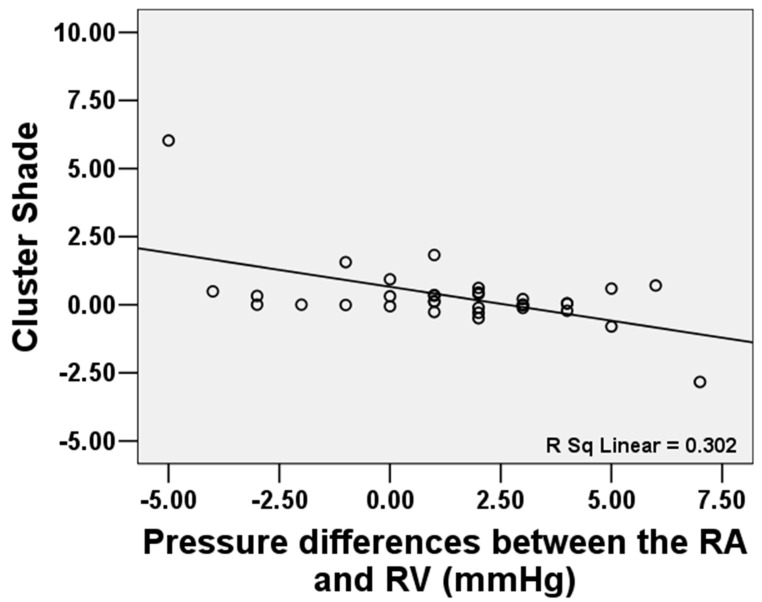
The pressure differences between the RA and RV were related to “Cluster Shade” (GLCM) (r = −0.55, *p* = 0.001).

Among 33 patients with FTR, mean RA pressure was associated with “Auto correlation” (GLCM) (r = 0.429, *p* = 0.013), “Joint Average” (GLCM) (r = 0.411, *p* = 0.017), “Maximum Probability” (GLCM) (r = 0.411, *p* = 0.017), “High Gray Level Emphasis” (GLDM) (r = 0.412, *p* = 0.014), “Small Dependence High Gray Level Emphasis” (GLDM) (r = 0.408, *p* = 0.018), “High Gray Level Run Emphasis” (GLRLM) (r = 0.409, *p* = 0.018), and “Short Run High Gray Level Emphasis” (GLRLM) (r = 0.402, *p* = 0.021). Mean RV pressure was related with “Interquartile Range” (First order) (r = 0.471, *p* = 0.006), “Skewness” (First order) (r = −0.478, *p* = 0.005), “Auto correlation” (GLCM) (r = 0.414, *p* = 0.017), “Joint Average” (GLCM) (r = 0.401, *p* = 0.021), “Maximum Probability” (r = 0.401, *p* = 0.021), “High Gray Level Emphasis” (GLDM) (r = 0.402, *p* = 0.021), and “Large Dependence High Gray Level Emphasis” (GLDM) (r = −0.446, *p* = 0.009). The pressure differences between the RA and RV were related with “Cluster Shade” (GLCM) (r = −0.55, *p* = 0.001). See Figure 3.

In total, good intra- and interobserver agreements were found on 86 (92%) and 82 (88%) radiomic features, respectively.

## 4. Discussion

In the present study, we demonstrated that multiple radiomic features acquired from the RA blood pool using a fixed-size ROI are associated with the existence of FTR as well as pressures and pressure gradient among the right heart chambers. Cine MRI-derived radiomics has demonstrated its potential to describe complicated interplay among the atrium, valve, and ventricle in the context of PH and FTR.

PH is a progressive condition characterized by elevated pressure in the pulmonary arteries and the right heart resulting from various underlying causes. The severity of FTR is a direct marker of RV dysfunction, an ominous sign in PH, as RV failure is the leading cause of morbidity and mortality in PH patients [13]. Hence, identifying the existence and severity of FTR provides therapeutic guidance; for instance, significant FTR may influence decisions regarding the timing of specific interventions, such as tricuspid valve repair or PH-specific therapies.

A commonly used MRI-based method for quantitatively assessing valve regurgitation relies on an indirect approach. This method can be used to calculate regurgitant fraction (RF) on the mitral valve by dividing the mitral regurgitation volume (MRvol) by the LV stroke volume (LVSV). This method can be performed on both echocardiography and MRI. When MRI is used, MRvol is determined as the difference between the left ventricular stroke volume (LVSV) and the aortic forward flow (AFF). LVSV is derived from regular short-axis cine balanced steady-state free precession (bSSFP) images, while AFF is derived by using phase-contrast velocity mapping (PCM), separately. Gelfand et al. enrolled 141 consecutive patients (age 53 ± 15 years; 57% male) with mitral regurgitation (MR) and aortic regurgitation (AR) who performed MRI and echocardiography with a short interval (median, 31 days). The authors found that there is good agreement between severities of MR and AR graded by MRI and color Doppler [14]. However, Uretsky et al. investigated 38 patients who underwent mitral valve surgery for treating regurgitation. The authors found poor agreements between MRI and echocardiographic estimates of MR severity. Furthermore, there was a strong correlation between MRI-derived MR severity and post-surgical LV remodeling (r = 0.85; *p* < 0.0001), but no significant correlation between echocardiography-derived MR severity and post-surgical LV remodeling (r = 0.32, *p* = 0.1) [15]. However, MRI-derived calculation for RF is complicated and time consuming, and its usage has not been validated in assessing FTR.

Recently, new MRI methods have been applied for the quantification of FTR. Driessen et al. assessed hemodynamics in 21 healthy volunteers and 67 patients with RV pressure-load using both 4D-flow MRI and echocardiography. The authors found that the tricuspid flows derived by the two methods are highly correlated [16]. Another straightforward method to detect valve regurgitation is solely based on cine MRI (acquired with SSFP technique) data. In theory, the blood pools in cardiac chambers on SSFP images are “bright”. However, the “bright signal” can be “contaminated” by transverse spins within the cardiac chamber, which are brought on by turbulence. Valve regurgitation can be a significant source of turbulence. Sometimes, significant regurgitation flow can be seen as a “dark area” on cine images because of the transverse spins brought on by turbulence resulting from regurgitation. Reddy et al. reported using a semiquantitative system to grade the severity of valve regurgitation with a 5-point system (0 to 4, trace, mild, moderate, moderate-to-severe, and severe) based on visual observation of SSFP cine images. The authors found that visual assessment of regurgitant severity is accurate and reproducible as compared with quantitative assessment [6]. However, the detection of subtle changes in “dark” or “bright” in SSFP image using the “naked eye” can be arbitrary and challenging.

Radiomics can be used to detect and quantify signal changes in images with greater sensitivity than the “naked eye”. Our previous studies have demonstrated that cine MRI-derived radiomic features of cardiac blood pool and great vessels can represent altered cardiac motion and its consequent hemodynamics changes [7,8,9]. In the present study, we detected the differences in multiple radiomic features within the RA during ventricular systole between PH patients with and without FTR. Since the RA can be enlarged under PH, we used a fixed-size ROI to exclude the influence of increased RA volume or area. These differences could be mainly attributed to the signal “contaminations” caused by the existence of FTR, and are significant enough to be used to discriminate PH patients with and without FTR. Our findings further expand the application of regular cine MRI-derived radiomics to assess valve regurgitation without requiring additional scans, such as PCM.

Our study has limitations. First, we were unable to compare the “accuracy” of radiomic features to other MRI-based methods, such as 4D-flow MRI or RF, in detecting and grading FTR due to the retrospective nature of this study. However, future research is warranted to explore the prognostic value of radiomic features in tracking FTR progression and guiding appropriate treatments. Second, we did not compare this method to visual evaluation of FTR on multiple cine views because four-chamber is the only standard view in regular cine to observe the RA. Third, to avoid overfitting in multiple comparisons, we investigated only 93 basic radiomic features in this “proof-of-concept” study. Larger-scale studies are needed to evaluate the clinical relevance of high-dimensional features, such as those derived from wavelet transformations, in representing FTR.

## 5. Conclusions

Cine MRI-derived radiomic features of the cardiac blood pool differ between PH patients with and without FTR. Cine MRI shows promise as a method for assessing valvular regurgitation in the context of complex cardiovascular syndromes.

## Figures and Tables

**Table 1:** Demographic data and structural, functional, and hemodynamic measurements of 53 PH patients with and without FTR.

	With FTR (*n* = 33)	Without FTR (*n* = 20)	*p* Values
Age (years)	60.5 ± 13.1	56. 6 ± 14.6	0.324
Male (%)	16 (49)	7 (35)	0.4
Postcapillary PH (%)	15 (42)	11 (55)	0.577
Height (cm)	179.4 ± 10.5	166.2 ± 7.2	0.128
Weight (Kg)	94.8 ± 25.1	87 ± 19.7	0.245
BMI	32.3 ± 7	31.5 ± 7	0.664
LVEDV (mL)	146.1 ± 58.4	150.8 ± 53.2	0.77
LVESV (mL)	64.8 ± 39.4	76.8 ± 50.7	0.349
LVSV (mL)	81.3 ± 28.8	74.1 ± 18.1	0.323
LVCO (L/min)	5.6 ± 2	5.1 ± 1.4	0.375
LVEF (%)	57.5 ± 11.2	53 ± 14.7	0.225
LVM (g)	104.5 ± 25.7	101.7 ± 37.8	0.758
LVCI (L/min/m^2^)	2.7 ± 0.8	2.6 ± 0.7	0.654
RVEDV (mL)	179 ± 55.7	141.8 ± 28.5	0.008 *
RVESV (mL)	100.5 ± 45.8	71.8 ± 25	0.013 *
RVSV (mL)	78.4 ± 25.8	70 ± 20	0.22
RVCO (L/min)	5.4 ± 1.8	4.8 ± 1.3	0.237
RVEF (%)	45.6 ± 12.4	49.9 ± 11.4	0.218
RVCI (L/min/m^2^)	2.6 ± 0.8	2.4 ± 0.6	0.405
sPAP (mmHg)	62.8 ± 18.6	54.6 ± 18.1	0.122
dPAP (mmHg)	25.2 ± 9	22.8 ± 7.5	0.31
mPAP (mmHg)	38.7 ± 10.1	34.1 ± 10.5	0.119
PCWP (mmHg)	15.4 ± 6.4	16.9 ± 6	0.395
RAP (mmHg)	10.4 ± 4.1	9 ± 4.6	0.222
RVP (mmHg)	11.8 ± 4.3	12.6 ± 4.6	0.522
PVR (wood unit)	5.2 ± 2.9	3.8 ± 2.8	0.089

* With significant differences.

## Data Availability

The data supporting the results of the present study are available from the corresponding author under a “data sharing agreement.
